# Microbial Stimulation and Succession following a Test Well Injection Simulating CO₂ Leakage into a Shallow Newark Basin Aquifer

**DOI:** 10.1371/journal.pone.0117812

**Published:** 2015-01-30

**Authors:** Gregory O’Mullan, M. Elias Dueker, Kale Clauson, Qiang Yang, Kelsey Umemoto, Natalia Zakharova, Juerg Matter, Martin Stute, Taro Takahashi, David Goldberg

**Affiliations:** 1 School of Earth and Environmental Sciences, Queens College, City University of New York, Flushing, New York, United States of America; 2 Lamont-Doherty Earth Observatory, Columbia University, Palisades, New York, United States of America; 3 Department of Environmental Science, Barnard College, New York, New York, United States of America; 4 National Oceanography Centre, University of Southampton, Southampton, United Kingdom; University of Kansas, UNITED STATES

## Abstract

In addition to efforts aimed at reducing anthropogenic production of greenhouse gases, geological storage of CO_2_ is being explored as a strategy to reduce atmospheric greenhouse gas emission and mitigate climate change. Previous studies of the deep subsurface in North America have not fully considered the potential negative effects of CO_2_ leakage into shallow drinking water aquifers, especially from a microbiological perspective. A test well in the Newark Rift Basin was utilized in two field experiments to investigate patterns of microbial succession following injection of CO_2_-saturated water into an isolated aquifer interval, simulating a CO_2_ leakage scenario. A decrease in pH following injection of CO_2_ saturated aquifer water was accompanied by mobilization of trace elements (e.g. Fe and Mn), and increased bacterial cell concentrations in the recovered water. 16S ribosomal RNA gene sequence libraries from samples collected before and after the test well injection were compared to link variability in geochemistry to changes in aquifer microbiology. Significant changes in microbial composition, compared to background conditions, were found following the test well injections, including a decrease in Proteobacteria, and an increased presence of Firmicutes, Verrucomicrobia and microbial taxa often noted to be associated with iron and sulfate reduction. The concurrence of increased microbial cell concentrations and rapid microbial community succession indicate significant changes in aquifer microbial communities immediately following the experimental CO_2_ leakage event. Samples collected one year post-injection were similar in cell number to the original background condition and community composition, although not identical, began to revert toward the pre-injection condition, indicating microbial resilience following a leakage disturbance. This study provides a first glimpse into the *in situ* successional response of microbial communities to CO_2_ leakage after subsurface injection in the Newark Basin and the potential microbiological impact of CO_2_ leakage on drinking water resources.

## Introduction

Global water scarcity and the reduction of industrial CO_2_ emissions are considered to be two of society’s major environmental management challenges for this century [1]–[7]. Among currently proposed CO_2_ mitigation techniques, geological storage (GS), accomplished by injection of CO_2_ into deep geological formations, is one of the most promising alternatives [8–13]. Progress with implementation of this technology has been slowed, in part, by concerns over poorly understood alterations to subsurface water resources. In addition to the potential alteration of groundwater geochemistry, CO_2_ leakage from deep formations to shallow drinking water aquifers has the potential to alter microbial communities by reducing pH and introducing, directly or indirectly, alternative substrates for microbial growth.

Microbial life in the subsurface is thought to represent a globally significant reservoir of biodiversity that remains largely unexplored [14–15]. Initial characterizations of subsurface microbial communities suggest that geochemistry has a controlling influence on microbial community structure (e.g. [16]) but it is also known that microbial communities have an influence on the rate of geochemical reactions (e.g.[17]). In a survey of the bacterial communities in drinking water wells, temperature and iron concentration were determined to be controlling factors of community structure and composition [18]. Even slight increases in Fe concentrations resulted in modified bacterial communities and promoted the growth of iron oxidizing bacteria, which are known to foul well pumps and degrade water quality [18]. A similar survey across the pristine Mahomet aquifer of east-central Illinois found that sulfate concentration was an important indicator for the balance between sulfate reducers and iron reducers as dominant components of the microbial community [19]. Interactions between microbes and geochemistry remain poorly constrained in most natural systems targeted for geological sequestration of CO_2_ and the in-situ successional response of microbes in shallow drinking water aquifers to a CO_2_ leakage event is still poorly constrained.

The experiment reported in this paper was designed to simulate an unintended leakage or migration of dissolved CO_2_ into a shallow drinking water aquifer after CO_2_ injection for the purpose of geological storage. Once in the subsurface, the injected CO_2_ may be transformed by geochemical reactions controlled by the chemical composition of the formation water, pressure, temperature, rock mineralogy [20–22], and microbial biogeochemistry [23]. As a result of these reactions and physical mixing the formation fluid would be initially acidified, followed by a potential neutralization. Subsequent leakage of the acidified formation fluid into shallower drinking water aquifers would be expected to shift pH and, presumably, lead to succession of microbial community structure and metabolism. Onstott [23] predicted rapid successional patterns linked to altered availability of electron donors and acceptors, including the potential for increased microbial metal reduction and methanogenesis. Previous laboratory-based investigations suggest that exposure to CO_2_ results in initial declines in bacterial abundance, but survival and adaptation of a stress resistant component of the community [24]. The CO2SINK project, an in situ CO_2_ injection designed to systematically monitor the microbial response to elevated concentrations of CO_2_, observe a shift from sulfate reducers to methanogens following a high pressure CO_2_ injection into a saline aquifer [25–26].

Bacterial taxa can respond quite differently to changes in pH, with methanogens displaying broad tolerance to pH variability [27], while other bacterial genera like *Acidothiobacillus* have growth optima at low pH, as seen in acid mine drainage [28]. Strong selective pressures favor broad tolerance to acidic conditions for some pathogens, such as *E. coli*, which are known to have diverse physiological adaptations that allow resistance to intestinal pH ranging from 2.0 to 7.0 [29]. Many of these pathogens prefer to grow at near neutral pH, such as those generally found within aquifers, but can persist and remain infective under variable pH [30], potentially allowing them to rebound from a subsurface pH disturbance more quickly than many other bacterial taxa.

Despite the potential for large changes in microbial composition and metal mobilization in aquifer drinking water, the microbially mediated biogeochemistry in response to elevated CO_2_ remains understudied compared to engineering and geochemical constraints [22] and to date there have been no *in situ* assessments of the microbial response to CO_2_ leakage into drinking water aquifers. To begin bridging this gap, we conducted the first microbial characterization of Newark Basin subsurface bacteria from a shallow drinking water aquifer subjected to repeated CO_2_ leakage scenario experiments. Using large-scale 16S rRNA gene sequencing from aquifer water samples collected during the two field injections, we attempted to address the following questions: 1) Are there observable microbiological responses to geochemical changes in a shallow drinking water aquifer following GS and potential CO_2_ leakage?; 2) What are the successional responses of the aquifer microbial communities?; and 3) Is it possible to identify microbial groups, over-represented in the early- and mid-phases of the leakage experiment, whose changing abundance may act as useful indicators for acidification from GS and potential CO_2_ leakage in the Newark Basin?

## Methods

### Study site and injection experiment

**Description of study site and injection experiment**. Located in Palisades New York (41.0039°N, 73.9126°W), the Lamont-Doherty Earth Observatory test well (TW-3) penetrates the Palisades Sill and extends into the underlying sedimentary rock formations of the Newark Basin. The Newark Basin interbedded sedimentary layers are abundant in iron oxide minerals and include sandstone, siltstone, and mudstone [31]. The Palisades Sill, a diabase intrusion, is approximately 230 m thick at this location, capping metamorphosed sedimentary formations [32–33]. The injections for this study were targeted to a low transmissivity (0.023 m^2^ day^-1^) permeable zone at 364 m with no detectable ambient flow, allowing for an appropriate duration for experimental incubation of CO_2_ saturated waters within the formation.

As described by Yang et al [34], two push-pull experiments were conducted into an isolated interval at 362–366 m depth in the test well during summers of 2011 and 2012. The interval was isolated by an inflatable packer system, and for several days prior to each injection experiment, formation water was pumped out at a flow rate of ~ 3.5 L min^-1^ to estimate interval transmissivity and characterize background conditions of the interval. During these pumping tests, continuous sensor measurements were made for pH, specific conductance (SC), dissolved oxygen (DO), and oxidation-reduction potential (ORP). While characterizing background conditions the pumped formation water was also used to extensively rinse three previously cleaned polyethylene tanks at the test well site, and then 3,400 liters of the formation water was captured in the tanks for acidification with CO_2_ and mixing with chemical tracers for injection. Once in the tanks, the formation water was slowly bubbled with CO_2_ for 16 hours to achieve saturation at one atmosphere partial pressure and Potassium Bromide (KBr) was added to a final concentration of 45.53 mg L^-1^ as a tracer. The KBr tracer allowed hydrogeological flow and mixing patterns of injection water with background formation fluid to be constrained, improving interpretation of geochemical and microbiological dynamics during the *in situ* experiment.

Approximately 3,050 liters of the CO_2_ acidified aquifer water was then injected from the holding tanks back into the isolated interval, at a flow rate of 4.6 L min^-1^ over an 11 hour period to simulate leakage, or unintended migration, of fluids from a GS site into a shallow drinking water aquifer. The CO_2_ bubbling of fluid in holding tanks continued during the injection to maintain CO_2_ saturation. Immediately following injection of the acidified fluids, 70 liters of unaltered aquifer water, that had been temporarily held in a fourth polyethylene tank without CO_2_ bubbling, was injected as a “chaser” to clear the injection tubing and to ensure full injection of the 3,050 liters of acidified aquifer fluid into the targeted subsurface interval. The 70 liter chaser was approximately twice the volume of the injection tubing. The injection interval, between 362 and 366 m depth, is more permeable radially than the intervals above and below and remained sealed by the packer system, eliminating vertical exchange within the test well, during the entirety of the background aquifer water pumping, injection, *in situ* incubation, and extraction “pump-back” sampling. The pressure above and below the packed off interval within the well was monitored during injection, incubation, and pumping to ensure that flow did not re-enter the well from another interval, but no pressure anomaly was detected, suggesting that the injected fluids remained within the formation injection interval.

To simplify description and analysis of the data, both experiments have been divided into experimental phases labeled “Background”, “Early”, “Mid”, and “Late”, which corresponded to the ratio of the volume of water pumped (Vp) out versus the volume of water injected (Vi) into the aquifer (as per Table 1). “*Background*” samples were collected pre-injection while water was being pumped out of the test interval prior to acidification via CO_2_ bubbling, but only after environmental parameters including pH, dissolved oxygen (DO), and oxidation-reduction potential (ORP) stabilized for the pumped aquifer fluid. “*Early*” samples were taken post-acidification and post-injection, during the initial extraction pump-back and while the ratio of volume pumped/volume injected was less than 1. “*Mid*” samples were taken during the later stages of pump-back when the ratio was greater than 1 but less than 10. “*Late*” samples were taken after the ratio was greater than 10.

**Table 1:** Organization of samples and 454 pyrosequencing data by phase of the experiment.

Injection	Phase	Vol. pumped/ Vol. injected	# Samples	# Bacterial sequences	# Archaeal sequences	# Total sequences
Injection 1	Background	Pre-injection	2	14,982	896	15,878
	Early	<1	3	18,253	2,933	21,186
	Mid	>1, <10	3	19,937	1,030	20,967
	Late	>10	3	13,582	372	13,954
Injection 2	Background	Pre-injection	3	19,351	1,399	20,750
	Early	<1	4	16,272	9,706	25,978
	Mid	>1, <10	6	38,791	12,945	51,736
	Late	>10	2	9,249	1,828	11,077
Total			26	150,417	31,109	181,526

During the 2011 experiment, an initial sample was collected seven days after the injection to measure the tracer concentration, pH, and specific conductance, by pumping to extract approximately 90 liters water. This sample was considered part of the “Early” phase, even though continuous pump-back had not begun. Before any discrete sample was collected and evaluated by sensor or laboratory processing, a volume greater than the internal volume of the injection/recovery tubing was purged, so that only fluids incubated in the test interval were evaluated. This small volume discrete sample at 7 days was intended to create minimal disturbance to the *in situ* incubation, but it allowed an initial evaluation of the mixing and transport of injected fluids in the test interval so that a decision could be made about the appropriate duration of *in situ* incubation before continuous pump-back. It was decided that the *in situ* incubation, prior to continuous pump back of the interval fluids, would last for 20 days after the injection. This time interval was viewed as a balance between providing adequate time for microbial and geochemical succession to occur and also permitting adequate recovery of injected fluids and avoiding complications from extensive mixing and dilution with waters surrounding the test zone.

Twenty days after incubation, recovery of injected fluid began with continuous pumping at 2.6 L min^-1^ for 33 days, equivalent to 40 times the injected volume, consisting of the injected fluids mixed with background interval water. Measurement of the conservative KBr tracer allowed the fraction of initial injected fluid versus background water to be evaluated in the recovered fluids. During this pump-back 20 sets of samples were collected to characterize geochemical alteration and microbial succession. It should be noted that dilution of the injected fluid, geochemical alteration (e.g. pH), and length of the incubation are correlated factors in this experiment. A repeat of the experiment occurred in the summer of 2012, following the same procedure, however, the *in situ* incubation lasted for 40 days to allow additional time for microbial succession following the injection disturbance, and the continuous pump-back lasted for 30 days.

**Ethics Statement**. Authorization for the injection experiment was obtained from the United States Environmental Protection Agency Underground Injection Program (UIC ID: 04NY08707045). No animals or endangered species were involved in this research.

### Geochemical and microbiological sample collection

Geochemical sampling was conducted as described by Yang et al [34]. Briefly, groundwater was filtered through 0.45 μm membranes for major anions, filtered and acidified to 1% HNO_3_ (Fisher Optima) for major cations and trace elements, and unfiltered water was used for Br^-^ tracer analysis. A YSI multi-parameter meter was used with a flow through cell to monitor temperature, pH, SC, ORP and DO concentrations throughout the injection experiments.

Samples for microbiological analyses were collected simultaneously with the geochemical samples using sterilized collection containers. At each sampling point, 50 ml aliquots were formaldehyde-preserved (3% final concentration) in sterile tubes and stored at 4°C until microbial concentrations were microscopically ascertained. Approximately 10 L of aquifer water were passed through duplicate 0.2 μm “sterivex” cartridge filters (Millipore, Darmstadt, Germany) that were immediately flash-frozen in liquid nitrogen and then stored at-80°C until molecular analyses were performed. One L of unfiltered water was transported to the laboratory, in the dark and on ice, to allow for measurement of Fecal Indicator Bacteria (FIB) within 6 hours of collection.

### Microscopic cell count, DNA concentration, and fecal indicator bacteria

To characterize the microbial response to CO_2_ leakage after GS, microbial cell concentrations were determined for each sample through the course of the injection experiments. Cell abundances were microscopically determined from formaldehyde fixed aquifer samples using SYBR Green staining, according to Noble et al [35]. The concentration of DNA extracted from samples (see below) was used as a second, independent, method of estimating microbial abundance in the samples to evaluate the microbial dynamics and to confirm patterns observed with microscopic cells counts. DNA was extracted from “sterivex” filters using the PowerWater DNA isolation kit (MoBio Laboratories, Carlsbad, CA), according to manufacturer’s instructions. DNA concentration of extractions and subsequent amplifications were determined using a Qubit fluorometer (Invitrogen, Grand Island, NY, USA). Enumeration of the FIB, *E. coli* and *Enterococcus*, were performed from 100 ml of unfiltered water using IDEXX enterolert and colilert selective media (www.idexx.com). FIB were enumerated to test for contamination with surface water and also because, as a commonly used indicator of drinking water quality, the response of FIB to CO_2_ leakage events was of interest.

### Microbial community successional dynamics

**DNA amplification and sequencing**. Bacterial primers 8F (5′-AGRGTTTGATCCTGGCTCAG-3′) and 1492R (5′-CGGCTACCTTGTTACGACTT-3′) [36] were used with 35 PCR cycles of 45 seconds of denaturation at 94°C, 45 seconds of annealing at 55°C, and 1 minute elongation at 72°C, followed by gel electrophoresis, to initially confirm that extracted DNA was PCR-amplifiable and to ensure that DNA-free controls did not yield amplification product. Subsequently, bacterial and archaeal community composition were determined using amplicon pyrosequencing performed at Molecular Research DNA labs (www.mrdnalab.com, MRDNA, Shallowater, TX, USA), using a protocol described by Dowd et al [37] which has been utilized in describing a wide range of environmental and health related microbiomes [37–39]. Briefly, parallel sequencing reactions were prepared from each DNA extraction using both the eubacterial primer 27F and archaeal primer 349F. Amplification was performed through a single-step 30-cycle PCR using HotStarTaq Plus Master Mix Kit (Qiagen, Valencia, CA). PCR was performed under the following conditions: 94°C for 3 minutes, followed by 28 cycles of 94°C for 30 seconds; 53°C for 40 seconds and 72°C for 1 minute; after which a final elongation step at 72°C for 5 minutes was performed. All amplicon products were then mixed in equal concentrations and purified using Agencourt Ampure beads (Agencourt Bioscience Corporation, MA, USA.) and sequenced using Roche 454 FLX titanium instruments and reagents, following manufacturer’s guidelines. Sequence read files and associated sample data are available from the National Center for Biotechnology Information database (www.ncbi.nlm.nih.gov/sra) under Bioproject PRJNA258542, including Sequence Read Archive accession numbers SRX729863 and SRX732154 to SRX732204.

**Analysis of DNA Sequence Data**. To generate a high-quality dataset for analysis, DNA sequences were processed with the Mothur platform [40–41]. PyroNoise was used allowing for 1 mismatch to the barcode and 2 mismatches to the forward primer. Sequences were then trimmed to remove barcodes and primer sequences, and sequences with less than 200 bp and/or homopolymers greater than 8 bp were removed from downstream analyses. Remaining sequences were aligned using SILVA reference alignments and chimeras were detected and removed using UCHIME [42]. Sequences were then taxonomically classified using the Mothur-formatted version of the Ribosomal Database Project training set [43], at 80% cutoff, then binned by genus to create phylotype OTU’s.

The PC-ORD software package (version 4.01; MJM Software) [44] was utilized for multivariate analyses of microbial community and environmental data. Before analysis, OTU data were relativized for sampling effort, as is commonly required in microbial ecology [45], by dividing the number of sequences for an OTU by the total number of sequences from the sample, creating a relative frequency for each OTU. Environmental data were normalized by dividing each variable by the maximum observed value across samples, placing all environmental values on a relative scale of zero to one. Environmental factors that initially contained negative values, such as ORP, had a number equal to the most negative value observed from any sample added before normalization to avoid inclusion of negative values that can be incompatible with some downstream analyses.

Hierarchical cluster analysis [46] was conducted for both environmental factors and community composition using Sorenson (Bray-Curtis) distance and farthest neighbor group linkage to examine the similarity of samples. Environmental factors used in the clustering analysis included: pH, specific conductance, ORP, pCO_2_, sulfate, manganese, and iron. A Mantel test [47] was used to compare the structure in the observed data to 1,000 randomizations of the same dataset, allowing distance matrices from environmental factors and community composition to be examined for non-random structure. A Multi-Response Permutation Procedure (MRPP) was used to test for differences among *a priori* selected groups, corresponding in this case to samples grouped by phase of the experiment (i.e. “Background”, “Early”, “Mid” and “Late”; Table 1). Non-metric Multidimensional Scaling (NMS) was used to visualize the similarity of samples based on DNA sequence composition analyzed at the level of genera and to examine patterns in the groups of samples corresponding to phase of the injection experiment. A NMS Scree plot was used to determine that the ordination was optimized using three dimensions, with significantly lower NMS stress (p = 0.0196) for the observed data compared to 1,000 randomizations of the data set, with the majority (0.887) of variation described by these three axes. Indicator Species Analysis, also with samples grouped based on phase of injection, was used to detect genera of specific interest for future environmental monitoring for GS [48]. The value of an OTU as an indicator for a particular phase of the experiment was determined by the proportional abundance of an OTU relative to the abundance of the OTU in all other groups examined. The statistical significance of these indicator values is determined by comparison of observed indicator values to indicator values resulting after 1,000 randomizations of the data set.

## Results

### Injection experiment and geochemical context

**Fig 1:**
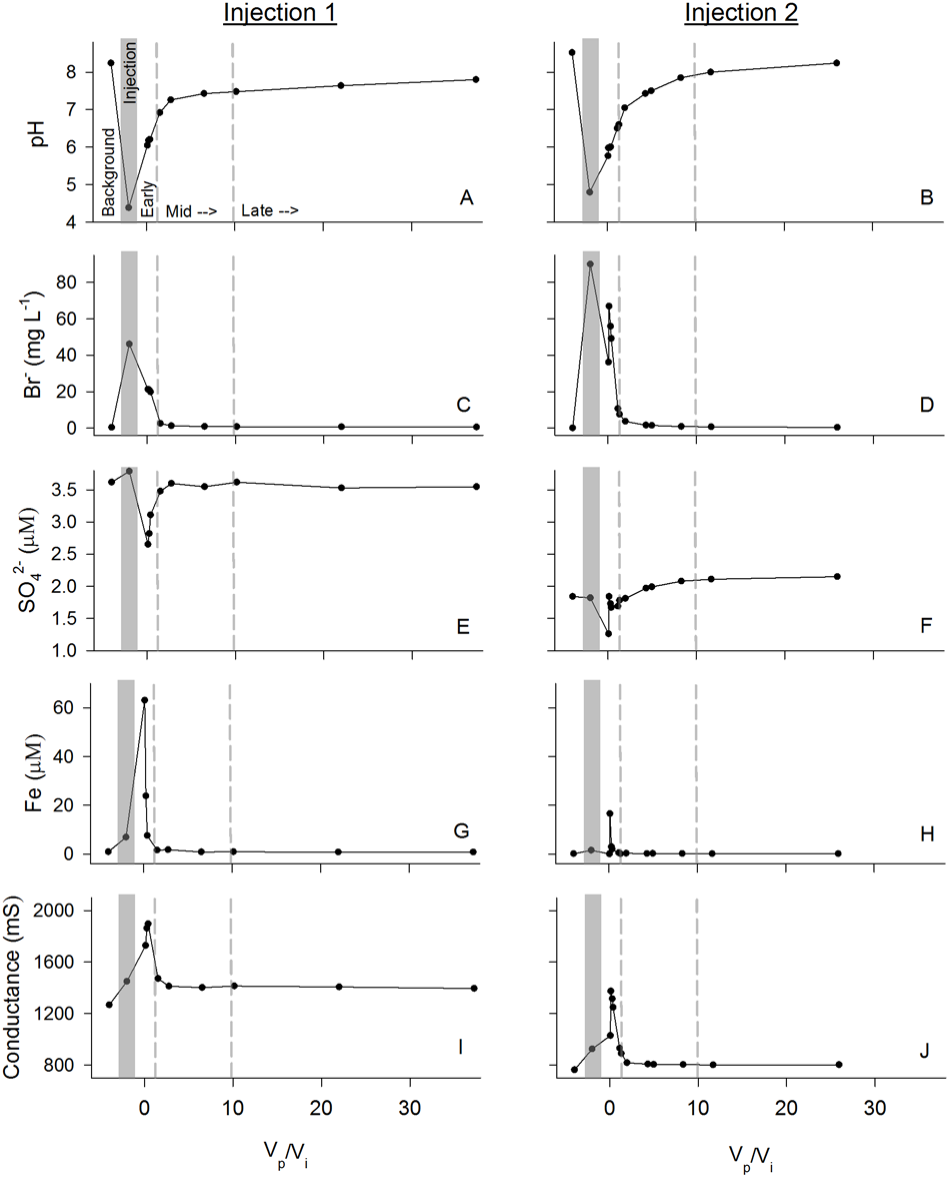
Chemical and physical parameters. Chemical and physical parameters over the course of the in-situ injection experiment V_p_/V_i_ = Volume pumped/Volume injected, with greater ratios representing longer incubation and greater dilution of injection fluids. Injection 1 on the left (A, C, E, G, I), and Injection 2 on the right (B, D, F, H, J). Dashed and shaded lines on x-axis separate: “Background” aquifer fluid prior to bubbling with CO_2_; “Injection” fluid sampled during the injection, following CO_2_ bubbling but prior to in situ incubation; and “Early”, “Mid”, and “Late” phases of the in situ experiment, with longer incubation represented by a higher ratio of volume pumped to volume injected (see Table 1).

CO_2_ injection into the aquifer produced clear geochemical responses during both field injection experiments. Background aquifer water showed a Na-SO_4_ water type. Pre-injection bubbling with CO_2_ in storage tanks did not change major ion concentrations or alkalinity, but resulted in an increase in the total dissolved CO_2_ (= [H_2_CO_3_] + [HCO_3_^-^] + [CO_3_^=^]). During both experiments, Background (pre-CO_2_ bubbling) samples were slightly basic (pH 8.2–8.5) (Fig. 1a,b), and microaerobic (DO = 1.0–1.5mg L^-1^). Injection of the CO_2_-saturated water (pH of approximately 4) resulted in an initial sharp decrease in aquifer pH, with an eventual return to pre-injection conditions (Fig. 1a,b). The KBr tracer allowed estimation of the mixing ratio of the injected high CO_2_ water with aquifer water as well as the total percent recovery of the injected water during pump-back extraction phases (Early, Mid, and Late). Recovery of injected water was estimated to be 73% in the Injection 1 experiment and 79% in the Injection 2 experiment, with the KBr concentration approaching zero (Fig. 1c,d), and injected fluid estimated to contribute less than 1% of the concentration in the final Late phase fluid samples. Water samples collected after injection, during the Early extraction phase of the experiment (pumped/injected volume ratio < 1), demonstrated major geochemical alterations, with notable increases of HCO_3_^-^, alkalinity, Ca, Mg and Si concentrations, increased by 2–12 times over Background phase concentrations. Sulfate initially decreased by 20–30% (Fig. 1e,f) while both Fe (Fig. 1g,h) and Mn [34] immediately increased in concentration during both experiments along with an initial doubling of conductivity, which also returned slowly toward pre-injection conditions (Fig. 1i,j).

### Microbiological response to geochemical change

**Fig 2:**
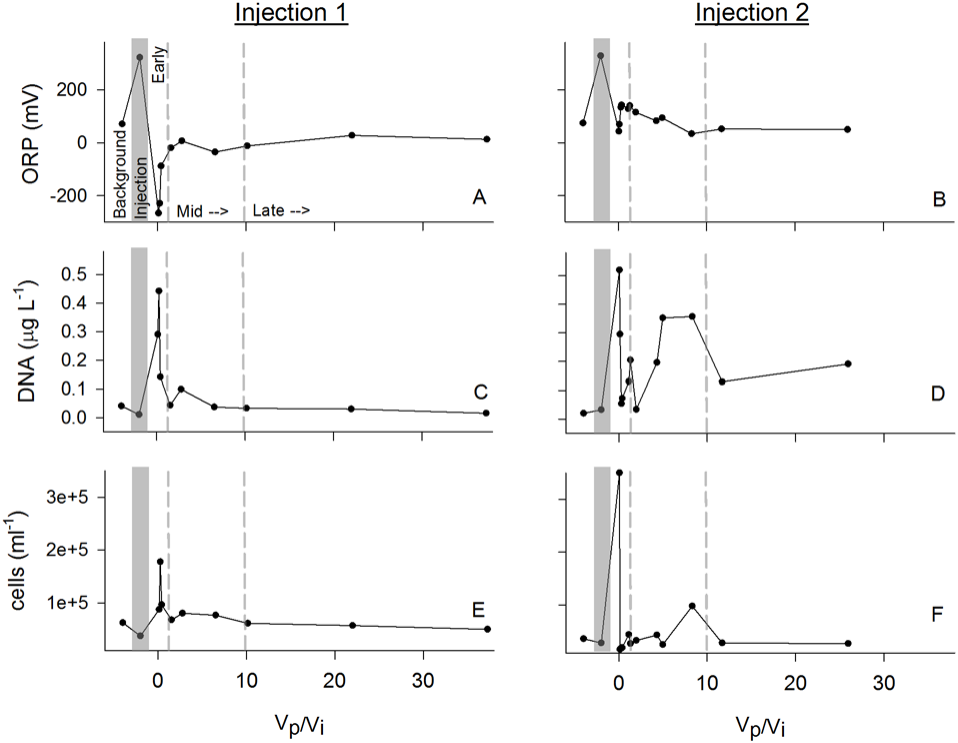
Biological parameters. Biological parameter concentrations over the course of the in-situ injection experiment. V_p_/V_i_ = Volume pumped/Volume injected, with greater ratios representing longer incubation and more greater dilution of injection fluids. Injection 1 on the left (A, C, E, G), and Injection 2 on the right (B, D, F, H). As in Fig. 1, dashed and shaded lines on the x-axis separate: “Background” pre-injection conditions; “Injection” fluids prior to in situ incubation; “Pumpback” of fluids following in situ incubation during the Early, Mid, and Late phases.

As geochemical conditions, such as pH, changed in the early phase of the experiment, it was expected that microbial communities would respond to these alterations, providing potential biogeochemical interactions with metabolically relevant factors such as ORP (Fig. 2a,b), Fe (Fig. 1g,h), and sulfate (Fig. 1e,f). Background microbial cell concentrations were low in both injection experiments with a mean of 4.0 x 10^4^ cells ml^-1^, and decreased slightly during CO_2_ bubbling and injection, but spiked significantly after injection (to 2.0 x 10^5^ in Injection 1 and 3.5 x 10^5^ in Injection 2). These concentrations then returned to background concentrations (Fig. 2e,f) in the later stages of recovery. The concentration of cells and DNA extracted from samples (Fig. 2c,d) were positively associated. Assuming 2 fg of DNA per cell [49], the pattern of microbial abundance estimated by microscopy and DNA concentration were in close agreement for both injections, displaying very similar dynamics that confirm an initial peak in microbial abundance in the early phases of recovery that approached background concentrations in later phases of the experiment (Fig. 2c,d).

After a quality check (see methods) of 454 sequencing results, 150,417 bacterial sequences and 31,109 archaeal sequences were maintained for downstream taxonomic analyses (Table 1). These sequences revealed a diverse and dynamic microbial community in aquifer water over the course of both injection experiments, including 633 OTUs that were identified at the genus level for most downstream analyses. Hierarchical cluster analyses were performed using both environmental factors and microbial community composition (S1 Fig. and S2 Fig.). Cluster analyses of environmental factors demonstrated distinct clusters for Early phases of the experiment (S1 Fig.), while cluster analyses of microbial communities demonstrated distinct clusters for both Background and Early phases of the experiment, indicating substantial microbial succession following the initial injection disturbance. A mantel test detected significant (r = 0.373; p = 0.001), non-random, structure between distance matrices from microbial community composition and environmental conditions, supporting an interaction between environmental conditions and microbial composition in this *in situ* aquifer disturbance experiment.

### Microbial successional dynamics

**Fig 3:**
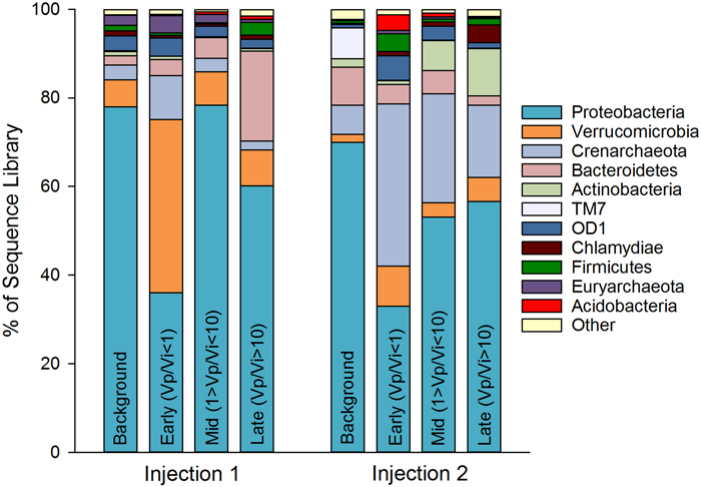
Phylum level classification. Phylum-level classification of bacteria and archaea in aquifer water during the four phases of the CO_2_ injection experiments: Background, Early, Mid, and Late. V_p_/V_i_ = Volume pumped/Volume injected, with greater ratios representing longer incubation and more greater dilution of injection fluids.

**Fig 4:**
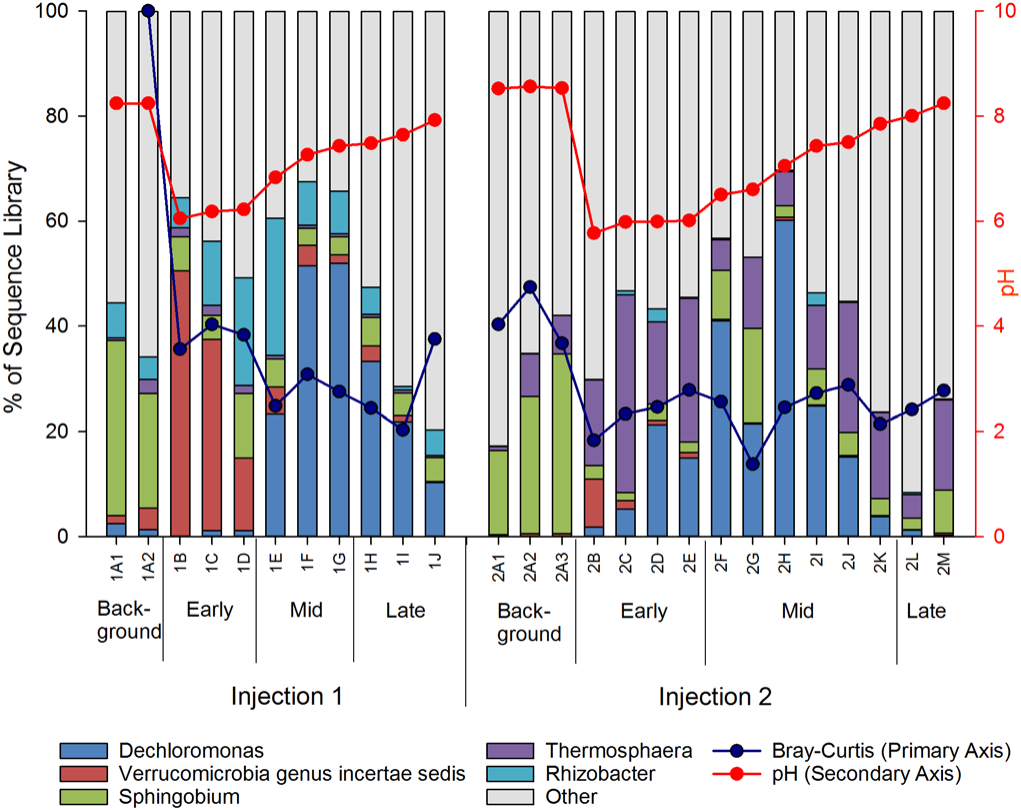
Succession of dominant genera. Succession of dominant genera in sequence libraries from samples collected during the injection experiments. Only genera representing >25% of sequences in at least one sample are considered dominant and labeled in the legend. Red line corresponds to pH, representing environmental change. The blue line corresponds to Bray-Curtis similarity, as compared to Background samples from Injection 1.

Phylum-level patterns of aquifer bacterial community alteration were similar in the two injection experiments (Fig. 3). Before both injections, background aquifer samples were dominated by Proteobacteria, but after CO_2_ injection Verrucomicrobia, Crenarchaeota and Bacteroidetes had increased representation in the sequence libraries. It is worth noting that in both injections, microbial communities did not return to pre-injection conditions on the phylum level even though the tracer concentration indicates that injected fluids constitute 1% or less of the final Late phase fluid. One year after the first injection disturbance, the Background condition in 2012 (Injection 2) was similar, although not identical, to the 2011 (Injection 1) Background community (Figs. 3 and 4), indicating some degree of microbial community resilience on longer time scales.

MRPP analyses, conducted at the level of genera, indicate that significant differences in community composition occurred among the four phases of the experiment (p < 0.01). Dominant genera (< 25% in any single sample) in aquifer waters changed dramatically after CO_2_ injection in both experiments (Fig. 4), with *Sphingobium* decreasing in relative abundance immediately after both injections and *Verrucomicrobia* increasing after both injections. *Dechloromonas* peaked, after a lag, in the Mid phases of both Injections 1 and 2, with a gradual decline in the Late phases. *Rhizobacter* was more dominant after Injection 1, while *Thermosphaera* had much higher relative abundances after Injection 2. Therefore, changes in the microbial community following injection were evident at both the levels of phyla and genera and similarity of samples to initial Background condition (prior to Injection 1), evaluated using the Bray-Curtis similarity index (Fig. 4), decreased post-injection in both experiments, demonstrating rapid community succession in response to the experimental disturbance.

**Fig 5:**
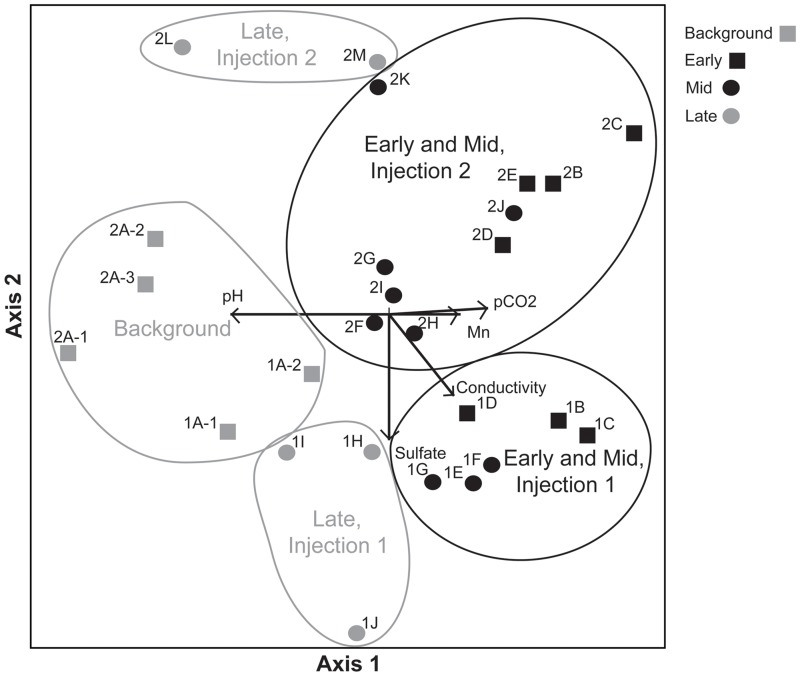
Non-metric Multidimensional Scaling (NMS) ordination. Non-metric Multidimensional Scaling (NMS) ordination of microbial community composition from the two injection experiments, representing the similarity and succession of samples. Samples are labeled with a number corresponding to injection 1 or injection 2, followed by a letter that corresponds to the collection order of samples with “A” representing background phase samples and consecutively collected samples listed in alphabetical order (See Fig. 4). Samples are grouped by “phase” of the injection experiment (see Table 1) with biplot overlay vectors (e.g pH, Sulfate) indicating the direction and relative magnitude of association between the environmental variables and the two axes used to ordinate community composition.

NMS (Non-metric Multidimensional Scaling) ordination of injection samples in OTU space (Fig. 5) displays patterns generally consistent with hierarchical clustering and MRPP analyses, and demonstrates the rapid change in microbial community composition that occurred in the Early and Mid phases of the injection experiment. Background samples form groups distinct from all other phases, most notably the Early phase samples following the injection disturbance. The community composition immediately following the 2011 and 2012 injection disturbances are similar (Figs. 4 and 5), with the ordination of Early and Mid phases demonstrating a shift across axis I, correlated with the changing pH for these samples. In contrast, samples collected from the Late phases of the two injections are more distinct from one another as compared to the Background samples, demonstrating a change in the microbial community that remained weeks after injection, even after >98% of the injected fluid had been recovered and environmental conditions such as pH and pCO_2_ had returned to near Background levels. The two experiments were performed in the same isolated aquifer interval approximately one year apart, and therefore, the Background sample from the 2012 injection represents a longer-term (one year) indication of succession from the prior year’s disturbance. While the background samples from the two injections are not identical, they do share the same dominant phylum (Proteobacteria, Fig. 3) and genus (Spingobium, Fig. 4) and group together in the NMS analysis and in hierarchical cluster analyses, indicating that the OTU composition of the microbial communities converge, reverting back toward the original Background assemblage, within one year of disturbance.

**Fig 6:**
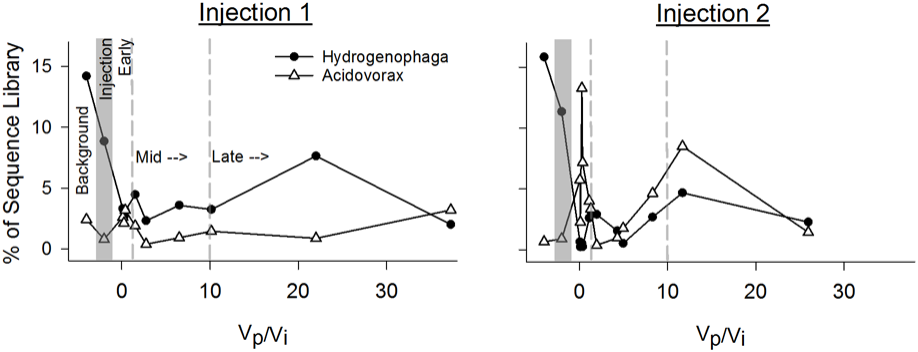
Hydrogen utilizers. Changes in two common hydrogen utilizing genera: A) Experiment 1 and B) Experiment 2. Vp/Vi = Volume Pumped/Volume Injected. As in Fig. 1, the x-axis is a ratio of the water volume pumped (Vp) out of the aquifer during the experiment and the CO_2_ saturated water volume injected (Vi) at the start of the experiment. Dashed and shaded lines on the x-axis separate: “Background”; “Injection”; “Early”; “Mid”; and “Late” phases of the in situ incubation.

**Fig 7:**
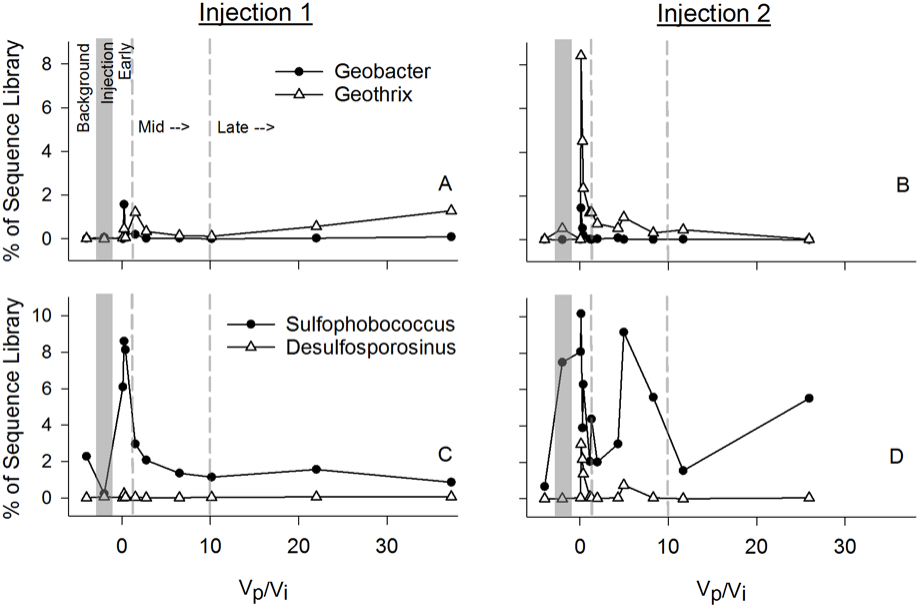
Iron and sulfate reducers. Relative importance of iron (A, B) and sulfate (C, D) reducers in sequence libraries from samples taken over the course of the injection experiments (Injection 1 (A, C) and Injection 2 (B, D)). Vp/Vi = Volume Pumped/Volume Injected. As in Fig. 1, the x-axis is a ratio of the water volume pumped (Vp) out of the aquifer during the experiment and the CO_2_ saturated water volume injected (Vi) at the start of the experiment. Dashed and shaded lines on the x-axis separate: “Background”; “Injection”; “Early”; “Mid”; and “Late” phases of the in situ incubation.

The patterns of succession summarized by NMS were also evident in the patterns of individual genera, with dominant components of the bacterial and archaeal communities shifting significantly post-injection. For instance, *Hydrogenophaga* and *Sphingobium*, dominant in both Background samples (15% and 30% of background sample sequence libraries, respectively), significantly decrease in representation in the sequence library post-injection (both < 5%, Figs. 4 and 6). *Dechloromonas*, *Verrucomicrobia*, *Thermosphaera*, and *Sulfophobococcus*, (all < 5% in background samples), become relatively more dominant (< 10%) in sequence libraries post-injection (Figs. 4 and 7).

**Fig 8:**
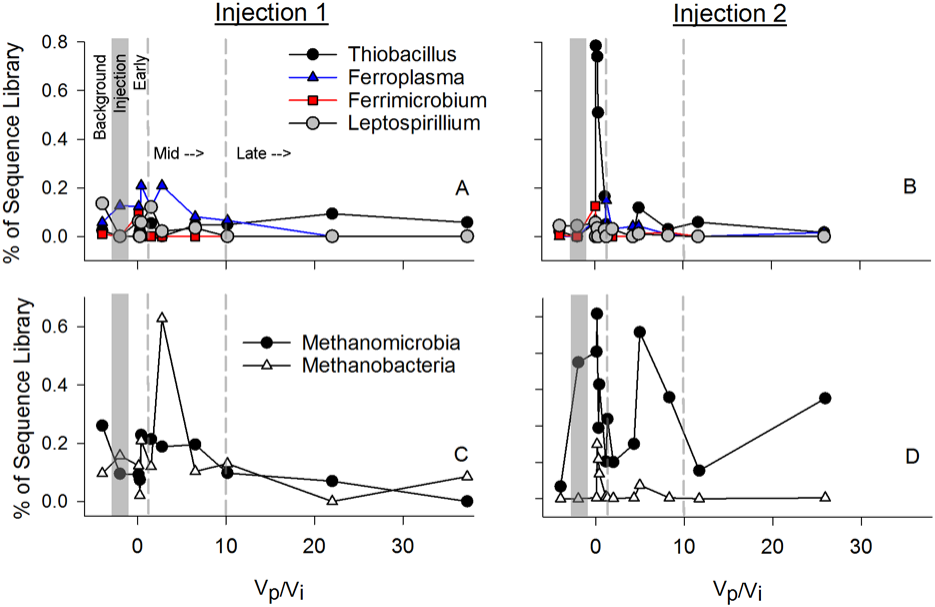
Sulfur oxidizers, iron oxidizers, and methanogens. Relative importance of sulfur and iron oxidizers (A, B) and methanogens (C, D) in sequence libraries from samples taken over the course of the injection experiments (Injection 1 (A, C) and Injection 2 (B, D)). Vp/Vi = Volume Pumped/Volume Injected. As in Fig. 1, the x-axis is a ratio of the water volume pumped (Vp) out of the aquifer during the experiment and the CO_2_ saturated water volume injected (Vi) at the start of the experiment. Dashed and shaded lines on the x-axis separate: “Background”; “Injection”; “Early”; “Mid”; and “Late” phases of the in situ incubation.

CO_2_ injection also increased representation of biogeochemically-relevant genera, including genera that have been associated with metal-reduction like *Geothrix*, *Geobacter*, *Desulfosporosinus*, and *Sulfophobococcus* (Fig. 7), which responded to CO_2_ injection, or the associated environmental changes, by sharp initial relative increases in library representation, then gradual return to near background levels in both field experiments. Genera associated with acid tolerance, and iron and sulfur oxidation, including *Acidovorax*, *Ferroplasma*, *Acidothiobacillus* and *Ferrimicrobium*, increased representation in sequence libraries immediately post-injection (Fig. 8), whereas *Leptospirillium* did not increase. Methanogens including *Methanomicrobia* and *Methanobacteria* also increased in library representation post-injection (Fig. 8).

An Indicator Species Analysis was conducted to evaluate the successional patterns from the perspective of future environmental monitoring for acidification and CO_2_ leakage into shallow drinking water aquifers due to GS. Twenty-nine genera were found to have significant (p-value < 0.05) use as indicators for the Early and Mid phases of the experiments, evident from their increased relative frequency in these phases (Table 2). Many of the possible indicators included genera previously associated iron and sulfate reduction, such as *Geobacter*, *Geothrix*, *Desulfosporosinus*, *Dechloromonas* providing additional evidence that microbial transitions following CO_2_ leakage events respond to changes in chemistry and would be expected to influence biogeochemical activity and potentially water quality.

**Table 2:** Genera found to have significance as microbial indicators for Early and Mid phase leakage conditions.

Genus	Phase of max. frequency	Indicator Value	p-value
Sulfophobococcus	Early	50.9	0.002
Sporomusa	Early	76.1	0.002
Paraeggerthella	Early	73.4	0.003
Veillonellaceae incertae sedis	Early	80.7	0.004
Sphingobacterium	Early	67.0	0.004
Verrucomicrobia 3 incertae sedis	Early	81.4	0.006
Nitriliruptor	Early	56.5	0.01
Paenibacillus	Early	62.6	0.01
GP6	Early	60.9	0.012
Solobacterium	Early	54.3	0.017
Thermomicrobium	Early	49.5	0.017
Desulfosporosinus	Early	73.8	0.019
Spartobacteria incertae sedis	Early	66.8	0.02
OD1 incertae sedis	Early	43.7	0.02
Coriobacterium	Early	42.9	0.021
Quadrisphaera	Early	52.1	0.022
Cupriavidus	Early	59.7	0.025
Delftia	Early	51.8	0.025
Hydrotalea	Early	49	0.025
Isosphaera	Early	53.7	0.027
Metylocystis	Early	42.5	0.033
Mariprofundus	Early	44.5	0.034
Geobacter	Early	75.3	0.034
Geothrix	Early	64.4	0.036
Thermogymnomonas	Early	46.2	0.042
Ferrimicrobium	Early	46	0.047
Dechloromonas	Mid	61.1	0.002
Ferruginibacter	Mid	79.4	0.007
Plesiocystis	Mid	55.3	0.049

Assays for viable FIB, *Enterococcus* and *E. coli*, were all negative indicating that the subsurface interval tested lacked any recent surface-associated sewage contamination. Similarly, enteric bacterial genera, such as *Escherichia*, *Enterococcus*, and *Enterobacter*, were either absent or extremely rare in all sequenced samples. We cannot adequately evaluate the successional response of enteric or sewage associated microbes to *in situ* acidification from this experiment and this remains an open question for future studies in contaminated drinking water aquifers.

## Discussion

### Observed disturbance from injection

Conditions in the Early and Mid phases of this field experiment, including pH, conductivity, sulfate, and Fe concentration (Fig. 1) [34], indicate highly altered geochemistry near the borehole injection interval. Based on prior laboratory [24], [50], [51] and field injection experiments [52–57] it was expected that water-rock interactions would quickly (on a timescale of hours to days) alter the chemistry of the injected fluids during incubation, including acid neutralization, mineral dissolution, and precipitation of carbonate minerals. As the CO_2_ saturated fluids were injected, the chemical changes induced by injected fluids presumably decreased with increasing distance from the well due to mixing with the aquifer water [52]. The spatial variability in the extent of disturbance was observed as temporal variability in our experimental data (comparing Early vs Late phases of pump-back). Similar geochemical gradients would be expected as CO_2_ impacted fluids migrated from the source of a hypothetical GS reservoir leakage event into a shallow potable water aquifer. The first recovered fluids (“Early” phase) had pH values as low as 5.7, while the injected fluids had a pH of approximately 4, indicating mixing and neutralization near the borehole. In the late phase of the experiment pH increased to near background levels, consistent with expectations and inversely related to the bromide tracer concentration (Fig. 1a-d). Major and trace elements including Ca, Mg, Si, Fe, Mn, Cr, Co, Ni, Cu, Zn, Rb, Sr, Ba, and U displayed rapidly increasing concentrations during the early phase of the experiment, as compared to background conditions [34], with release rates from the experiment similar to those observed in batch reactor experiments [58].

### Microbiological response to CO_2_ injection and altered geochemical conditions

Cell concentrations in Background water samples, estimated both microscopically and with DNA extractions (Fig. 2c-f), were low but comparable to measurements made in many other aquifers [19], [59–61]. Cell concentrations rose significantly in the Early phase of injection, potentially driven by altered metabolic substrates produced by the acidification disturbance, and then declined in the Mid and Late phases to approximately Background levels. ORP also decreased rapidly in the Early phase (Fig. 2a,b), likely as a response to the microbial stimulation indicated by rising cell counts, creating additional environmental variability that would cause microbial succession. The dropping ORP created conditions more favorable for reductive microbial metabolism and may have acted as a positive biogeochemical feedback on the observed metal mobilization (e.g. Fe) and reduction of sulfate.

The CO2SINK experiment [25–26] in northeast Germany examined the microbial response to a CO_2_ manipulation of a saline aquifer in siltstone, sandstone and mudstone of the Triassic Stuttgart Formation, which has a similar geological age to the Newark Basin formations, but is located at much greater depth (700–850 meters) below the surface. In Germany, the background cell concentrations were found to be approximately 10^6^ cells ml^-1^, noted to be at least an order of magnitude higher than most deep anaerobic aquifers previously measured [26]. Two days prior to CO_2_ injection, the CO2SINK test wells were flushed with N_2_ gas (referred to as “N_2_ lift”) to prepare them for CO_2_ injection. Cell counts were found to decrease by three orders of magnitude following the N_2_ lift and cell counts in the observation wells remained one order of magnitude below background levels for approximately 2 months following the subsequent CO_2_ injection [26]. This is in contrast to the results presented in this paper, where cell counts were found to initially increase (Early phase) compared to Background conditions. The difference may be due to the fact that the CO2SINK injection was simulating a high pressure, super-critical CO_2_ injection into a saline aquifer, while our experiment simulated leakage of diluted fluids from a CO_2_ storage reservoir into a shallower potable aquifer. Interestingly, the pH and metal mobilization response of both experiments were similar, despite the different microbial response.

Morozova et al [26] explain the decreased microbial abundances from CO2SINK as a cellular stress response expected due to acidification and the subsequent return to background levels as adaptation to these conditions [62–63]. Interpretation of the microbial abundance changes following the CO2SINK experiment is problematic because of the major cellular reduction following the N_2_ lift disturbance. Although Morozova et al [26] report the pattern as a decrease in cell abundances after CO_2_ injection, when compared to the abundances following the N_2_ lift disturbance, cell abundances in the Stuttgart formation in fact increased by more than an order of magnitude in the weeks following the injection of CO_2_. An increase in the percentage of active cells was reported in the months following injection. Abundances did not surpass the background, pre-injection levels, however. It is difficult to determine if the CO_2_ injection acted to stimulate the microbial community and increased the speed of recovery following the N_2_ lift, or if the CO_2_ injection acted in concert with the initial N_2_ lift disturbance to repress cell abundances below background levels. Based on the comparison of these studies it would be reasonable to expect that a high pressure, super-critical CO_2_ injection may initially cause repression of the microbial community, while a GS leakage scenario into a shallow potable aquifer may result in short term microbial stimulation. Both types of disturbances were found to cause microbial succession and point to the resiliency of the sub-surface microbial community.

In addition to changes in pH, CO_2_ chemistry, and cell abundance, the geochemical conditions in the Newark Basin injection shifted in other important ways including increased trace element concentrations (e.g. Fe) and lowered ORP, oxygen, and sulfate. These factors could be both a cause of microbial change and an effect of microbial change. For example, increased microbial abundance and activity would be expected to contribute to lowered oxygen and ORP. Similarly, increased microaerobic and anaerobic microbial activity, driven by decreased oxygen and ORP, would be expected to reinforce iron mobilization and sulfate depletion via microbial iron and sulfate reduction. While it is impossible to attribute these geochemical changes to biological activity alone, biogeochemical activity from stimulation of subsurface microbes is consistent with these geochemical patterns.

### Microbial successional dynamics and microbial indicators of CO_2_ leakage events

The Background bacterial community from the Newark Basin test interval, before CO_2_ injection, was dominated by Proteobacteria as has been observed in many shallow drinking water aquifers [18–19], [61], while the Archaea were dominated by Crenarchaeaota, similar to the pattern found by Lavalleur and Colwell [61]. The two dominant genera detected in background samples, *Sphingobium* and *Hydrogenophaga*, have also been previously detected in drinking water and pristine aquifers [18], [24], [61], [64–66]. *Sphingobium* is an obligately aerobic chemoheterotroph genus [64] and *Hydrogenophaga* is a facultative anaerobic chemoorganotrophic or chemolithoautotrophic genus capable of oxidizing hydrogen as an energy source using oxygen or nitrate as a terminal electron acceptor [67–68].

Proteobacteria remained the dominant phyla through the experiment despite the increase in relative abundance of Verrucomicrobia and Crenarchaeota following injection (Fig. 3). These data show that the subsurface microbial community can be rapidly altered in response to injection or leakage of CO_2_. The relative abundance of *Sphingobium* and *Hydrogenophaga*, decreased substantially following injection (Figs. 4 and 6), while *Dechloromonas* became the most abundant genus in the mid stages of recovery (Fig. 4). In addition to acidification stress, the decrease in *Sphingobium* may be due to their obligate aerobic metabolism, which would not be well suited to the decrease in oxygen and ORP following injection. It is less clear why *Hydrogenophaga* may be poorly suited to the conditions in the Early and Mid-phases of the experiment. Laboratory incubations simulating acidification of in situ fluids as part of the CO2SINK project also found *Hydogenophaga* to be abundant pre-CO_2_ acidification, but undetectable afterward [24].

Although the community composition from the deep saline fully-anoxic aquifer from the CO2SINK experiments was, not unexpectedly, quite different from the shallower Newark Basin potable aquifer, the importance of genera that have been associated with sulfate reduction was evident in both systems. The Stuttgart formation was dominated by fermentative halophilic bacteria such as *Halanaerobium*, and sulfate reducing bacteria such as *Desulfohalobium* and *Desulfotomaculum* [26], [69]. None of these genera were detected in the Newark Basin potable aquifer samples, perhaps due to their preference for higher salt environments. Genera associated with sulfur reduction, including *Dechloromonas*, *Desulfosporosinus*, and *Thermosphaera* did become dominant groups in the Early and Mid-phases (Figs. 4 and 7) following the drop in pH, oxygen, and ORP. Although the specific taxa vary between systems, as would be expected based on salinity differences, the functional group dynamics inferred from the 16S rRNA gene sequence data were similar.

Iron and metal reduction associated genera, such as *Geothrix*, *Geobacter*, and *Desulfosporosinus* (Fig. 7), also increased in relative abundance following acidification of in situ fluids in the Newark Basin experiment, indicating a transition expected under lower ORP and coinciding with the observed decreases in sulfate and increases in Fe and other trace metals. The peaks in iron and sulfate reducing genera tend to occur immediately after injection, while peaks in genera associated with iron and sulfur oxidation were often slightly delayed following injection (e.g. *Ferroplasma*, *Acidothiobacillus*; Fig. 7a,b and Fig. 8). Their abundance may be enhanced by the prior reductive activity, one of many possible syntrophic interactions. It is important to note that functionality of taxa was not directly measured and energetics alone should not be used to explain the microbial transitions, as the susceptibility to pH may be just as important in constraining successional patterns [26], [70], [71] and many of the genera peaking in the early and mid phases of injection are known to include acidotolerant or acidophilic taxa.

Finally, methanogenic genera, including *Methanomicrobia* and *Methanobacteria*, also increased in their relative abundance in the early- and mid-phases of the injection, coinciding with decreases in sulfate that have also been observed in other subsurface systems (e.g. [19], [59]). The peaks of methanogens were higher in 2011, when ORP was much lower than observed in the 2012 injection. Increases in both iron reducers and methanogens were predicted by prior modeling efforts of induced alterations due to GS [23] and were observed in the CO2SINK injections [26].

Indicator Species Analysis detected 29 genera whose relative abundance in sequence libraries increased significantly during the Early or Mid phases of injection (Table 2). These genera provide insights into the potential for metabolic transitions linked to geochemistry (e.g. increased iron and sulfur cycling) and provide possible targets for indicator organisms that could be used in GS leakage monitoring programs. It is interesting that the majority of these genera (26 of 29) were identified from the Early phase of the experiment, indicating that the largest changes in microbial composition would occur quickly after a leakage disturbance. In contrast, *Dechloromonas* displays a large increase post-injection, but peaks in the Mid phase, and becomes the most abundant genera. Other indicators of note include genera associated with iron and sulfur reduction such as *Geobacter*, *Geothrix*, and *Desulfosporosinus*.

The patterns of dominant genera (Fig. 4) and NMS analysis (Fig. 5) allow visualization of the changing microbial community, with similar patterns in the Background, Early and Mid phase communities across both experimental injections. In combination, these data demonstrate substantial and predictable succession in the microbial community, with increases in taxa matching the observed geochemical transitions and prior modeling efforts (e.g. [23]). The phylum and genus-level composition are consistent with a rapid transition from a background aquifer microbial community similar to those observed in low nutrient, pristine environments [60–61] to a microbial assemblage with increased representation of taxa previously found in acidified, metal rich environments (e.g. [72]). Rare organisms gained prominence post-injection, including some taxa associated with iron reduction, sulfate reduction, anaerobic iron oxidation and methanogenesis, demonstrating the potential for microbes to contribute to altered biogeochemical processes involved in trace gas production and metal mobilization.

### Potential significance for metal mobilization

Oxidative and reductive microbial metabolism can influence the mobility of metals, such as uranium or arsenic, and are therefore important for understanding potable water quality. For example, Senko et al [73] found that an acid-tolerant strain of *Desulfosporosinus* isolated from acid mine drainage reduced U (VI) in groundwater more rapidly at pH 4.4 than 7.1. Bioremediation strategies have relied upon microbially mediated reduction of U (VI) to insoluble U (IV) end products to reduce their mobility in groundwater [74], while increased ORP and dissolved oxygen have been associated with U oxidation and mobilization [75]. In the Early phase of the injection experiment, U concentrations were found to increase 100 fold as compared to Background levels, despite the lowering pH, DO, and ORP [34]. This pattern was attributed primarily to enhanced desorption caused by formation of uranyl carbonate complexes [34]. It is also possible that genera such as *Geobacter*, and *Geothrix*, which have been linked to the anaerobic oxidation of U [76] and were observed to increase in the Early and Mid phases, may also have influenced the observed U increase in this leakage scenario.

Although As concentrations were not observed to increase compared to Background conditions [34], microbes isolated from Newark Basin black shale have previously been shown to mobilize arsenic under sulfide oxidizing conditions [77]. Newark Basin black shales incubated in groundwater were quickly colonized with biofilms, and scanning electron microscopy detected bacteria shaped pits on pyrite crystals indicating bio-weathering [78]. Cultures from these surfaces were dominated by iron and pyritic sulfur oxidizers including *Geobacter* and *Dechloromonas* [78]. Both of these genera were abundant in our field experiment and peaked in Early or Mid phases (Table 2; Figs. 4 and 7) of the experiment, with *Dechloromonas* accounting for more than 50% of the detected sequences in some post-injection samples. This is not surprising as *Dechloromonas* has previously been identified in association with black shales in low pH environments [79]. The association of microbes with black shales from the Newark Basin may be of large biogeochemical significance, potentially impacting water quality through metal mobilization. Zhu et al [80] found that biologically active incubations of Newark basin black shale and its associated microbes, under sulfate reducing conditions, resulted in seven times more mobilization of arsenic than sterile controls. This supports the possibility that the microbial dynamics observed during the injection experiment could play a role in trace metal geochemistry resulting from a CO_2_ leakage event and could have important consequences for water quality in potable aquifers.

Many prior laboratory and modeling experiments studying CO_2_ injection or leakage associated metal mobilization have purposefully excluded or limited microbial activity (e.g. drying of samples in [49]), while other studies have stressed the importance of research that considers the microbial response and potential biogeochemical contribution [19], [23], [34], [61]. Although the duration of our field experiments is relatively short (weeks), disturbance conditions in a leakage scenario may also be short-lived, and the timescale of most metal mobilization is expected to be fast (days/weeks) [34], [50], [58]. Therefore, the rapid successional dynamics observed in this experiment may be well synchronized to the most important geochemical/biogeochemical alterations linked to metal mobilization. Even in experiments seeking to minimize biotic interactions, redox state was observed to be important in controlling metal mobilization [50], highlighting the potential for both direct (e.g. metal reduction) and indirect (e.g. lowered redox state and potential iron-sulfur interactions) influences of microbial activity on metal mobilization.

### Importance of free versus attached bacteria

It is worth noting that our methods did not allow for determination of the relative contribution of microbes attached to substrate vs. microbes suspended in aquifer water. In fact, our microbial methods only assessed the suspended microbial community, while the measured geochemistry is expected to integrate the activity of both the attached and suspended components of the community. In addition, our experiment did not characterize the microbial transitions that would occur from a non-CO_2_ saturated control injection, and the physical disturbance associated with injection may be important to consider. Previous research has determined that the percentage of microbial cells attached in aquifers is highly variable and poorly constrained, but that the majority of cells are often attached [15]. There is evidence that the abundance of unattached microbes may increase after aquifer disturbance [81] and that higher rates of well pumping can cause shearing of biofilms and increases in suspended cell counts [82–83]. Shearing of biofilms has also been speculated to account for some apparent shifts in microbial diversity or composition during groundwater experiments [84]. For example, Purkamo et al [66] speculate that increased concentrations of methanogens during a borehole experiment may be due to biofilm sheering. Apparent shifts in microbial composition due to biofilm sheering would be consistent with work by Flynn et al [19] demonstrating that the attached microbial communities contained a higher relative percentage of iron reducing bacteria, in comparison to the suspended community, which is also consistent with studies of iron oxidizers (e.g. [85]).

Prior to the injection experiment, thousands of liters were pumped from the well at a constant rate until stable background parameters were observed. In addition to stabilizing the background geochemistry, this pump-down procedure is likely to have removed, or decreased, the influence of variable biofilm sheering from our successional results. Rodan and Zachara [86] found that the rate and extent of iron reduction was correlated to oxide surface area, suggesting that changes in surface associations, surface area, or surface morphology could be important to both microbial composition and biogeochemistry. It is possible that injection disturbed biofilms, through either physical or geochemical interactions, resulting in a functional shift in microbial communities and surface detachment.

Tischer et al [81] showed that *Desulfosporosinus*, common in our post injection sequence libraries (Table 2; Fig. 7), were abundant in minicore samples acquired in the wall of a borehole. Flynn et al [19] found a distinct difference between attached and suspended microbial communities in pristine aquifer waters, with iron-reducing bacteria more abundant in attached communities than in suspended communities. The increase in genera associated with iron reduction in our post-injection samples may represent increased representation of attached communities in the samples and could be due to the shearing of biofilms from the borehole walls with pumping, the release of attached cells into aquifer water due to changes in conductivity or the increase of suspended iron reducing communities. It is possible that the observed microbial stimulation and succession instead demonstrate a shift from attached to suspended microbial communities. We are unable to differentiate between these responses in our data, although both are relevant considerations in the event of CO_2_ migration or leakage from GS reservoir.

## Conclusion

The microbial community from a Newark Basin test well was observed to display a large and significant shift in both microbial abundance and composition following an experimental injection of CO_2_-enriched aquifer water into a shallow potable water aquifer. This study represents the first experimental effort, using an *in situ* injection, to examine the microbial response to CO_2_ migration or leakage from a GS reservoir. The observed increase in cell counts, by nearly an order of magnitude, following the injection disturbance indicate either microbial growth and/or mobilization of microbes from surface-attachment to suspension in aquifer water, with either form of microbial stimulation relevant for influencing water quality in a leakage scenario. The microbial successional patterns in the Early and Mid phases, including apparent decreases in obligate aerobes (e.g. *Sphingobium*) and increase in genera often associate with both acid tolerance (e.g. *Acidothiobacilus*) and metal reduction (e.g *Dechloromonas*, *Geobacter*), demonstrate the potential for microbes to influence geochemistry and metal mobilization in aquifer waters following CO_2_ leakage. The early stages of succession were very similar in repeated injection experiments conducted one year apart in the same Newark Basin test interval and involved stimulation of a rare biosphere from the background community. In the one year period between experimental disturbances/injections, the microbial community was observed to shift back toward an abundance and composition more similar to the pre-injection background community. This suggests that the subsurface environment in Newark Basin is resilient and that low levels of CO_2_ leakage from a potential GS reservoir are unlikely to create a permanent, large-scale, alteration of the subsurface microbial community.

## Supporting Information

S1 Fig Hierarchical cluster analysis of environmental parameters measured during the two injection experiments. Environmental parameters used in the analysis were: pH, conductance, ORP, pCO_2_, Sulfate, Manganese, and Iron. Samples are labeled with a number corresponding to injection 1 or injection 2, followed by a letter that corresponds to the order of samples with “A” representing background phase samples and consecutively collected samples listed in alphabetical order (See Fig. 4).
(TIF)

S2 Fig Hierarchical cluster analysis of microbial community composition based upon DNA sequences identified to the level of genera during the two injection experiments. Samples are labeled with a number corresponding to injection 1 or injection 2, followed by a letter that corresponds to the order of samples with “A” representing background phase samples and consecutively collected samples listed in alphabetical order (See Fig. 4).
(TIF)
