# Correction: Microbial Stimulation and Succession following a Test Well Injection Simulating CO_2_ Leakage into a Shallow Newark Basin Aquifer

**DOI:** 10.1371/journal.pone.0121932

**Published:** 2015-03-26

**Authors:** The PLOS ONE Staff

There is a missing affiliation for Dr. M. Elias Dueker. M. Elias Dueker is affiliated with 1 School of Earth and Environmental Sciences, Queens College, City University of New York, Flushing, New York, United States of America, 2 Lamont-Doherty Earth Observatory, Columbia University, Palisades, New York, United States of America, and 5 Bard College, Annandale-on-Hudson, NY 12504.
