# Supplementary figures and images for: Microbial Stimulation and Succession following a Test Well Injection Simulating CO₂ Leakage into a Shallow Newark Basin Aquifer
Source: PLoS One. 2015 Jan 30;10(1):e0117812. doi: 10.1371/journal.pone.0117812 (PMC4312087; doi:10.1371/journal.pone.0117812)

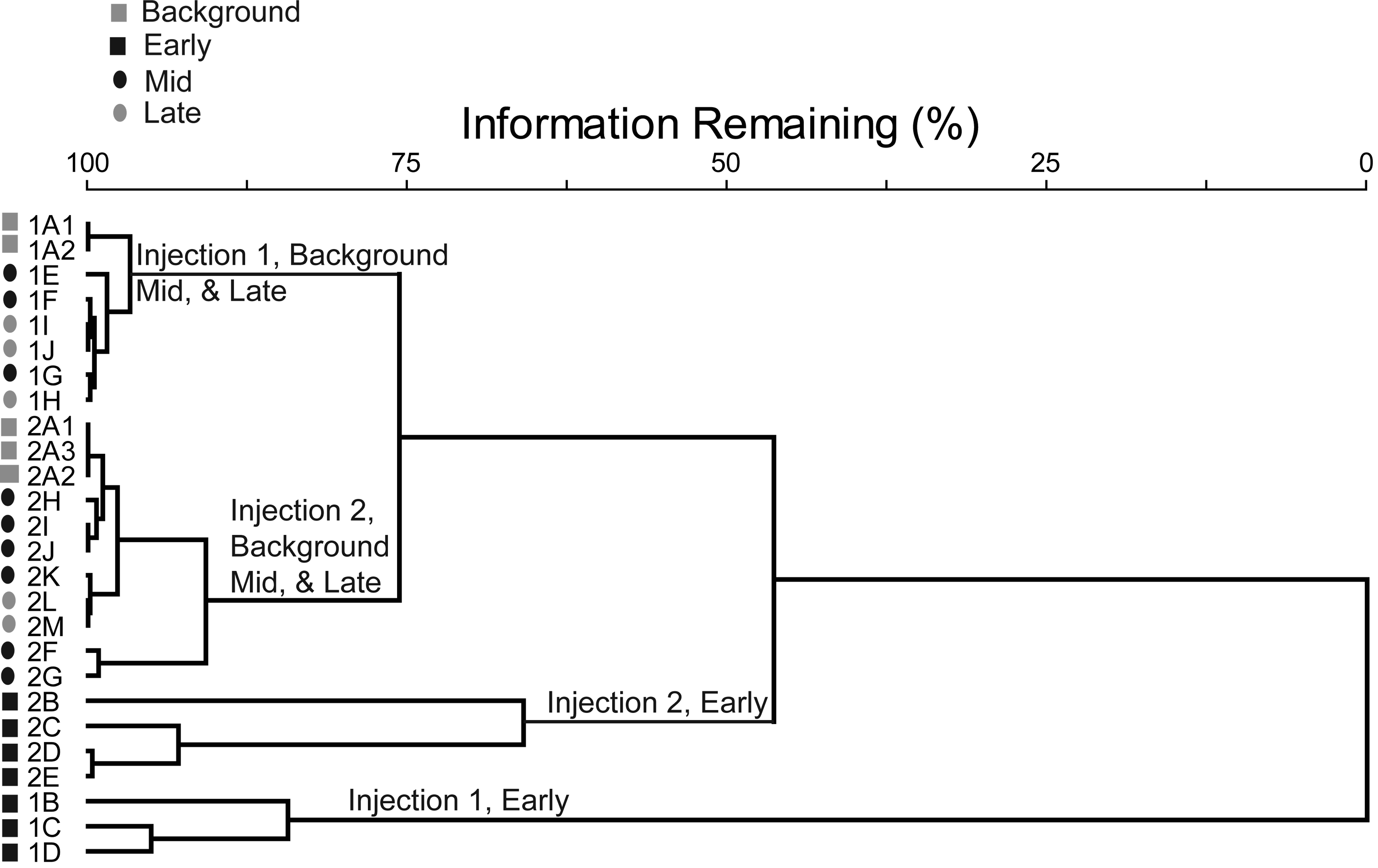

Supplement: S1 Fig — Environmental parameters used in the analysis were: pH, conductance, ORP, pCO2, Sulfate, Manganese, and Iron. Samples are labeled with a number corresponding to injection 1 or injection 2, followed by a letter that corresponds to the order of samples with “A” representing background phase samples and consecutively collected samples listed in alphabetical order (See Fig. 4). (TIF) [file pone.0117812.s001.tif]

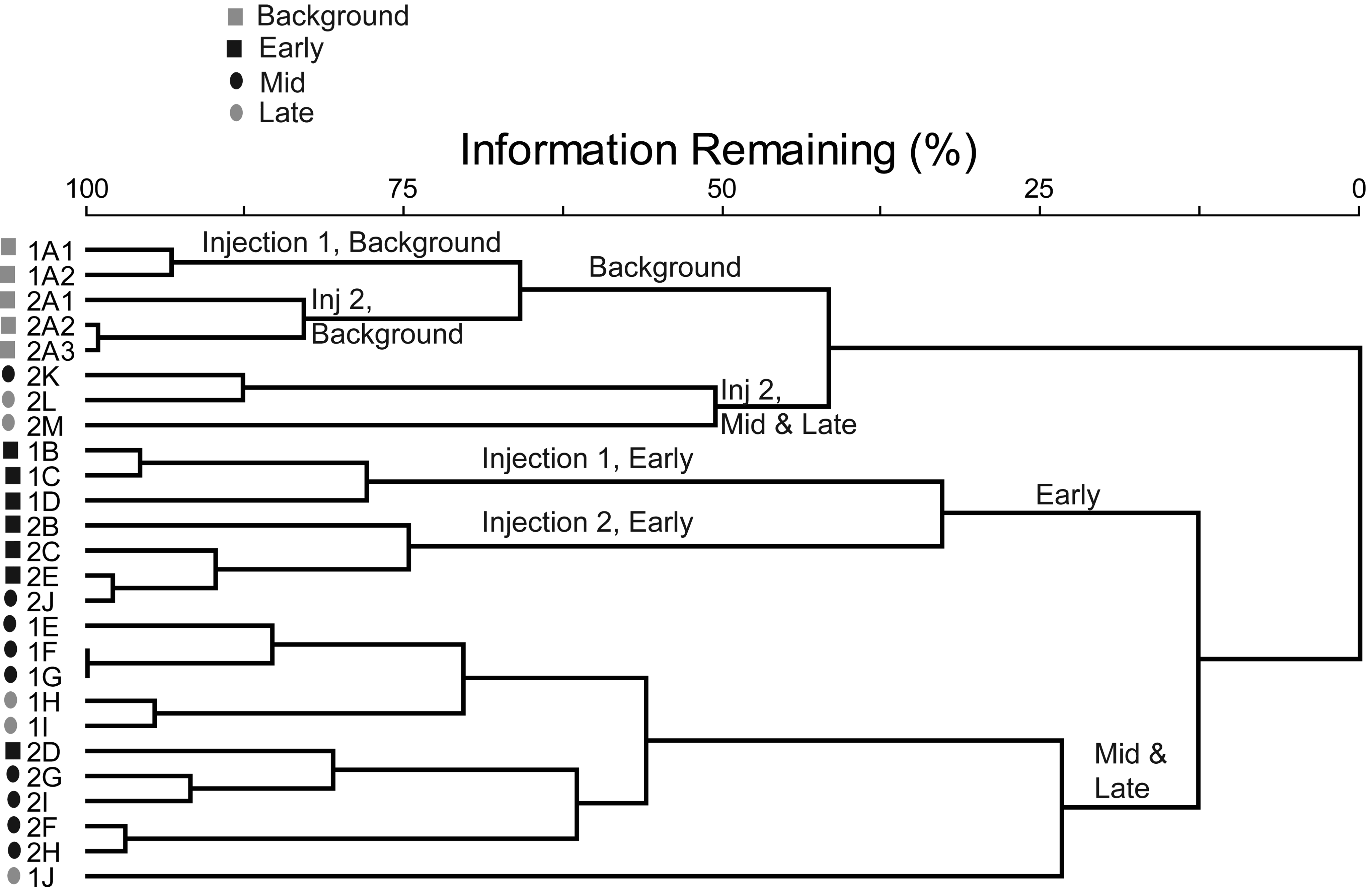

Supplement: S2 Fig — Samples are labeled with a number corresponding to injection 1 or injection 2, followed by a letter that corresponds to the order of samples with “A” representing background phase samples and consecutively collected samples listed in alphabetical order (See Fig. 4). (TIF) [file pone.0117812.s002.tif]
